# Psychopathy-related traits and the use of reward and social information: a computational approach

**DOI:** 10.3389/fpsyg.2013.00952

**Published:** 2013-12-19

**Authors:** Inti A. Brazil, Laurence T. Hunt, Berend H. Bulten, Roy P. C. Kessels, Ellen R. A. de Bruijn, Rogier B. Mars

**Affiliations:** ^1^Donders Institute for Brain, Cognition and Behaviour, Radboud UniversityNijmegen, Netherlands; ^2^PompestichtingNijmegen, Netherlands; ^3^Wellcome Trust Centre for Neuroimaging, University College LondonLondon, UK; ^4^Sobell Department of Motor Neuroscience, University College LondonLondon, UK; ^5^Department of Medical Psychology and Geriatrics, Radboud University Nijmegen Medical Centre, Donders Institute for Brain, Cognition and BehaviourNijmegen, Netherlands; ^6^Department of Clinical, Health, and Neuropsychology, Leiden Institute for Brain and Cognition, Leiden UniversityLeiden, Netherlands; ^7^Department of Experimental Psychology, University of OxfordOxford, UK; ^8^Oxford Centre for Functional MRI of the Brain, University of Oxford, John Radcliffe HospitalOxford, UK

**Keywords:** psychopathy, psychopathic traits, personality traits, individual differences, reinforcement learning, social learning, associative learning, computational modeling

## Abstract

Psychopathy is often linked to disturbed reinforcement-guided adaptation of behavior in both clinical and non-clinical populations. Recent work suggests that these disturbances might be due to a deficit in *actively using* information to guide changes in behavior. However, how much information is actually used to guide behavior is difficult to observe directly. Therefore, we used a computational model to estimate the use of information during learning. Thirty-six female subjects were recruited based on their total scores on the Psychopathic Personality Inventory (PPI), a self-report psychopathy list, and performed a task involving simultaneous learning of reward-based and social information. A Bayesian reinforcement-learning model was used to parameterize the use of each source of information during learning. Subsequently, we used the subscales of the PPI to assess psychopathy-related traits, and the traits that were strongly related to the model's parameters were isolated through a formal variable selection procedure. Finally, we assessed how these covaried with model parameters. We succeeded in isolating key personality traits believed to be relevant for psychopathy that can be related to model-based descriptions of subject behavior. Use of reward-history information was negatively related to levels of trait anxiety and fearlessness, whereas use of social advice decreased as the perceived ability to manipulate others and lack of anxiety increased. These results corroborate previous findings suggesting that sub-optimal use of different types of information might be implicated in psychopathy. They also further highlight the importance of considering the potential of computational modeling to understand the role of latent variables, such as the weight people give to various sources of information during goal-directed behavior, when conducting research on psychopathy-related traits and in the field of forensic psychiatry.

## Introduction

Adults and children with psychopathic tendencies typically show reduced affective-interpersonal functioning, often accompanied by an antisocial lifestyle (Hare et al., 1991; Viding et al., 2007; Sadeh and Verona, 2008; Verona et al., 2012). Research from our own and other labs has shown that offenders with high levels of psychopathic tendencies exhibit deficiencies in associative learning based on reward and punishment (Newman and Kosson, 1986; Budhani et al., 2006; von Borries et al., 2010). It has also been advocated that these deficiencies might lead to impaired associative learning based on social information, resulting in antisocial behavior and a lack of morality (Blair and Cipolotti, 2000; Blair, 2007; Brazil et al., 2011). This claim is also in line with findings in healthy individuals showing that associative learning of reward and social values follow the same mechanistic principles in the brain, albeit via separable neural substrates (Behrens et al., 2008, 2009).

Results obtained in our lab indicate that psychopathy seems to be related to a reduced ability to actively use information signaling that a change in current behavior is required in order to perform optimally (von Borries et al., 2010; Brazil et al., 2013). To date, however, there has been no direct quantification of how social and reward information is used during associative learning. One reason is that the mainstream experimental approaches in psychiatry do not allow the direct quantification of how much information is used to adapt behavior (see also Montague et al., 2012). However, this limitation can be overcome by incorporating computational modeling of behavior and known neurobiology in understanding psychiatric conditions (Huys et al., 2011; Maia and Frank, 2011; Buckholtz and Meyer-Lindenberg, 2012). Computational models of associative learning have proven to be increasingly helpful in explaining pathological behavior in neurological disorders like Parkinson's disease (Frank et al., 2004), but also in psychiatric disorders such as schizophrenia (Braver et al., 1999; Fletcher and Frith, 2008) and addiction (Redish et al., 2008). In these conditions, key model parameters can be related to specific aspects of these patients' impaired behavior (Frank et al., 2004) or neurobiology (Corlett et al., 2007), thus allowing the quantification of latent processes that are characteristic of these conditions (i.e., computational phenotypes) (Montague et al., 2012). However, this model-based approach has been notably scarce thus far in research into personality constructs with a less clear conceptual and neurocognitive background such as antisocial personality disorder and psychopathy (Blair, 2005; King-Casas et al., 2008).

There is an on-going debate about the conceptualization of psychopathy (see e.g., Lilienfeld et al., 2012; Miller and Lynam, 2012). Some scholars argue that psychopathy should be defined and assessed in terms of malicious characteristics (e.g., Hare, 2003; Neumann et al., 2012), while others believe that the definition should be broader to also include certain adaptive personality traits (Lilienfeld and Andrews, 1996; Patrick et al., 2009) and there is evidence supporting each approach. Lilienfeld and Andrews (1996) created a questionnaire assessing individual variations in eight common personality traits believed to be strongly related to key adaptive and maladaptive features of psychopathy. Further research suggests that the heightened presence of four of these personality traits may capture part of the aberrant interpersonal-affective personality characteristics and cognitive processing style typical to psychopathy relative to more generic antisocial (i.e. externalizing) personality profiles (see e.g., Poythress et al., 1998; Sadeh and Verona, 2008). The suggestion is that the typical traits are a lack of fear, reduced anxiety, guiltlessness/carelessness/lack of affiliative behavior, and social dominance/manipulative interpersonal style. However, there are very few studies directly relating individual differences in these traits to aspects of psychopathic personality profiles in a quantitative manner (see White et al., 2013).

The main goals of the present study were to use computational modeling to provide the very first direct quantification of the amount of information used to determine behavior during associative learning and to specify which psychopathy-related personality traits are linked to problems in using both social and non-social information. We reasoned that if the diminished *use* of information is a computational phenotype pertaining to psychopathy (relative to generic antisociality), it should also be present among the general population and be related to four personality traits argued to capture aspects of the affective-interpersonal dysfunctions linked to psychopathy and not to the other traits predominantly linked to generic antisociality. To achieve this we sampled a population with varying degrees of common personality traits linked to psychopathy (Lilienfeld and Andrews, 1996; but see Neumann et al., 2012). We then quantified the use of reward history and social advice information to guide behavior in an established reinforcement learning paradigm in which participants have to combine information from both sources to make optimal choices (Behrens et al., 2008) and used a variable selection method to identify the psychopathy-related traits with the most explanatory power.

## Methods

### Measure of psychopathy-related traits

Traits were assessed the Dutch translation of the Psychopathic Personality Inventory (PPI) [for more information see (Jelicic et al., 2004)], a self-report questionnaire used to index the presence of traits related to psychopathy in non-clinical samples (Sellbom et al., 2005). Higher scores correspond to higher impact of these traits on personality. The PPI consists of 187 items that are scored on a 4-point Likert scale. Each item loads on one of eight subscales, each subscale representing a different personality trait. The scales are Stress Immunity (displays reduced anxiety), Social Potency (is able to charm and manipulate others/is socially dominant), Fearlessness (lacks fear of harmful consequences), Machiavellian Egocentricity (is self-centered), Blame Externalization (blames others), Carefree Non-planfulness (lacks forethought), Impulsive Non-conformity (is reckless and unconventional) and Coldheartedness (is callous, guiltless).

### Participant recruitment

A large pool of potential participants was created through advertisements on a university website and on a national news website with a link to a digital version of the PPI (*N* = 485; 160 males and 325 females). The internal consistency of the subscales was acceptable (Chronbach's alpha = 0.71). Total PPI scores did not differ between males (*N* = 160, Mean = 343, *SD* = 39.9) and females (Mean = 350, *SD* = 38), indicating that scores were distributed equally between genders. Subsequently, total PPI scores were divided in quartiles, and participants were invited based on their scores. Participants from all quartiles (thus, from the entire range of PPI total scores) were invited to take part in the experimental session, but the top and bottom quartiles were oversampled in order to enhance the presence of extreme scores on both sides of the distribution (Bernat et al., 2011). The experimental sample initially consisted of a single, mixed-gender group of 40 individuals. Unfortunately, only 4 males were willing to participate leading to a strong gender imbalance within the group. Therefore, the male subjects were excluded from further analyses and the final sample consisted of 36 females (for PPI scores see Table 1), from which 22 (61%) belonged to the top and bottom quartiles of the selection pool and 14 to the 2nd and the 3rd quartile (39%).

**Table 1:** **Mean total PPI score and subscale scores for the experimental sample (*n* = 36)**.

**Variable**	**Mean (*SD*)**
Age	22.8 (6.4)
Total PPI score	336 (47.8)
Stress immunity	28.9 (5.4)
Social potency	56.6 (12.8)
Fearlessness	41.3 (10.0)
Coldheartedness	46.4 (7.3)
Blame externalization	31.8 (6.9)
Carefree non-planfulness	40.4 (5.7)
Machavellian egocentricity	54.6 (12.0)
Impulsive non-conformity	33.6 (6.3)

All participants received either course credits or a financial compensation and gave written informed consent. The study was approved by the local ethics committee of the Faculty of Social Sciences at the Radboud University in Nijmegen.

### Experimental task

**Figure 1:**
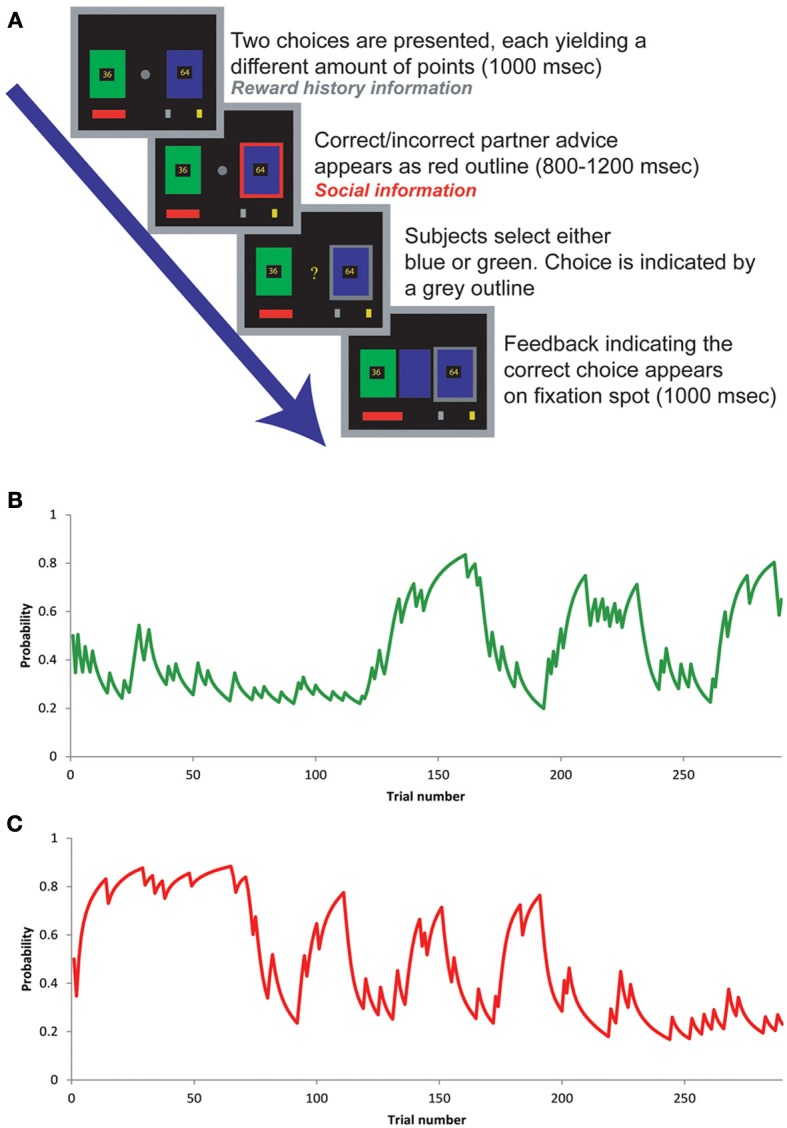
**(A)** Sequence of events and their timings during the experiment. **(B)** Probability of reward from choosing green card through the experiment. The line shows the probability estimated by the Bayesian reinforcement learning model. **(C)** The figure shows the model-derived probability of the confederate providing the correct answer through the experiment. Note that the model learns *independently* about both social and reward history information at the time feedback is received.

Completed 290 trials of a decision-making task in which they had to learn about the probability of receiving reward on two options (blue and green rectangles, Figure 1) (Behrens et al., 2008). Subjects repeatedly chose between the two rectangles in order to accumulate points. The number of points available (a random number between 1 and 100) was shown in the center of each rectangle; this number was added to the subject's score if the option was chosen and rewarded on that trial. Either blue or green could be correct on each trial, but the probability of the two colors being correct was not equal (*p*_blue_ = 1 − p_green_). The chance of each color being correct could be inferred based upon the recent outcome history, but was subject to reversals during the course of the experiment (see below). However, the reward magnitudes available were independent of the probabilities of each color being correct; thus, as a result of the difference in reward magnitudes associated with the blue and green options, subjects would sometimes choose to pick the less likely color if it was associated with a higher reward. Subjects saw a red bar onscreen, whose length depicted their current score; they aimed to reach a silver target to win €5, or a gold target to win €7.50.

Subjects simultaneously learnt about the reliability of advice from a social partner. On each trial, subjects received advice (red box around choice in Figure 1) about which rectangle to choose from a “human partner” (the experimenter), supposedly playing with them (in reality, the advice was computer-generated). The experimenter sat on the other side of a custom-made shield that divided the room, preventing any visual contact between the participant and the experimenter. Prior to the experiment, both “players” went through the instructions together. The partner's advice constituted what we refer to as the “social information” or “social advice” in the results. The partner's advice was predetermined prior to the experiment (and was, by design, uncorrelated with the reward history-based probability). A cover story was provided such that the partner might be incentivized to give either helpful or unhelpful advice in the experiment, and that this might change during the course of the experiment. In essence, participants were told that during the task the confederate's advice could be either correct or incorrect, but that the confederate was executing a different task and his advices were generated based on this task. Participants saw a demonstration of the task executed by the confederate when receiving the task instructions and they were told that the confederate held no knowledge of the participant's choices, nor whether green or blue was correct. That is, the confederate would provide advice and the computer (which were visibly connected through a cross-over network cable) would map this advice to the appropriate color [for further details see (Behrens et al., 2008)]. Irrespective of whether the advice was trustworthy or untrustworthy, the subject could exploit the advice to gain further information about which of the two options was the best choice on each trial. After the subject had responded (indicated by the gray box around the choice in Figure 1), the correct answer was revealed in the center of the screen, and was then replaced by a fixation point before the next trial began.

In summary, subjects had *three independent* sources of information available on each trial to guide their choices—(i) the magnitude of reward available on each option; (ii) the estimated probability of green/blue yielding reward, based on past experience; (iii) the estimated fidelity of the social partner's advice, based on past experience. The true (underlying) probabilities of both (ii) and (iii) were predetermined such that they varied independently of one another, and underwent several reversals during the course of the experiment (Behrens et al., 2008). This meant that subjects had to continually monitor and learn about each source of information throughout the experiment, and also that each source of information had unique explanatory power in explaining variation in choice behavior. Our key question focused on the degree to which subjects used (ii) and (iii) to guide their choices—a feature of their behavior that can be captured formally with a computational model.

### Modeling

**Figure 2:**
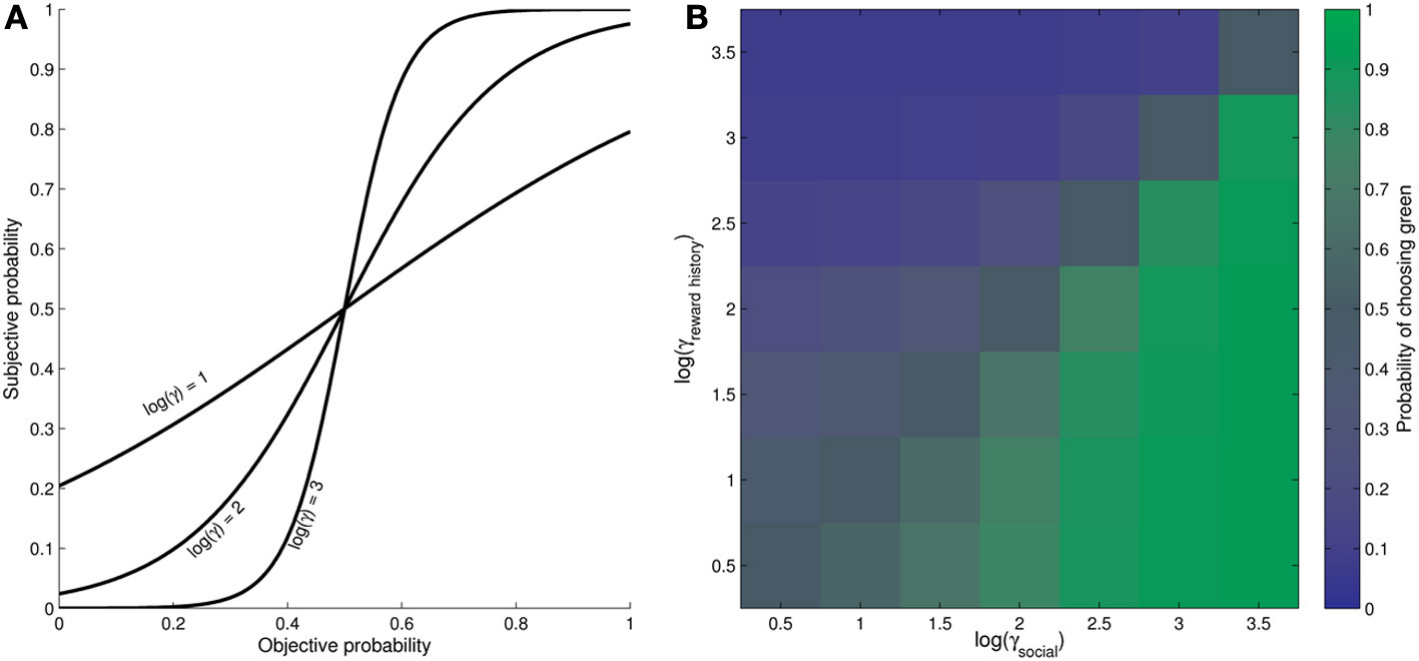
**Graphical depiction of the γ parameter in the model**. (See equations 1 and 2, section Mathematical model description, for algebraic description). **(A)** Example transform between objective (RL model-derived) probability and subjective probability, parameterized by γ. As γ increases, small differences in the “objective” probability (tracked by the model) are amplified to have a greater influence on subject behavior. **(B)** Posterior probability of choosing green for varying levels of γ_reward history_ and γ_social_, for one example trial, where reward history and advice are equally relevant, but suggest conflicting responses (reward history suggests blue choices, advice is to pick green). When γ_social_ = γ_reward history_ (diagonal), subject is equally likely to pick blue or green; when γ_social_ > γ_reward history_, subject is more likely to pick green; when γ_social_ < γ_reward history_, subject is more likely to pick blue. See section Modeling for details.

We fit a behavioral model to estimate the *influence* of each source of information on each subject's behavior (see mathematical description below). Based on behavioral and neuroimaging results from a previous study (Behrens et al., 2008), the model assumes that subjects use Bayesian reinforcement learning (RL) (Behrens et al., 2007) to track both the probability of green/blue being correct and the probability of receiving truthful advice, and then use this information to guide their behavior. The details of this Bayesian RL model are described in a previous paper (Behrens et al., 2007), and the resulting probabilities are shown in Figures 1B,C. The key feature of Bayesian RL is that it allows for a learning rate that *varies* depending upon the current stability or volatility of the environment (Yu and Dayan, 2005; Behrens et al., 2007). To capture the *extent* to which each subject used each source of information in guiding their choices, we fit a model that contains two parameters, γ_reward history_ and γ_social_, which have analogous functions for reward history and social information, respectively; importantly, these parameters are independent of the rate at which information is *learnt* in the task (which varies through the task via the RL model, and is not fit as a free parameter). The mathematical role of these parameters is described in equations 1 and 2 in section Mathematical model description, below. Intuitively, however, their role can be thought of as controlling the extent to which a given source of information influenced subject choices, as shown in Figure 2. If γ is high for a given source of information, then it means that the objective probability associated with that source of information is *amplified*, i.e., pushed more toward 1 if it is greater than 0.5, and more toward 0 if it is less than 0.5 (e.g., the steepest line in Figure 2A). Conversely, if γ is low, the objective probability is pulled toward 0.5, and so has less influence (e.g., the shallowest line in Figure 2A). We estimated these parameters (and a further temperature parameter β, capturing choice stochasticity) separately for each subject (see below), in order to investigate cross-subject variability in their expression.

The magnitudes of γ_reward history_ and γ_social_ then become important when we *combine* the sources of information to obtain an *overall probability of selecting green* on each trial. This is illustrated in Figure 2B, where we show the effect of varying the two parameters on the eventual probability of the subject wanting to select green for an example trial. In this trial, there is a 0.3 probability of green being rewarded given the recent reward history. However, the confederate has advised green, and there is a 0.7 probability that the confederate will give good advice. Hence, these two sources of information would cancel one another out—but *only* if the subject uses each source of information equally (i.e., γ_reward history_ = γ_social_). Conversely, if γ_social_ > γ_reward history_, then the subject will favor the social information and become more likely to pick green (green area in Figure 2B), whereas if γ_reward history_ > γ_social_, the subject will become more likely to pick blue (blue area in Figure 2B). Note that for simplicity, we have shown an example where the points on green and blue are equal; however, further interactions occur with the number of points available as these vary from trial to trial, and also as the probabilities of social and non-social information fluctuate independently of one another. In particular, subjects with small values of γ_reward history_ and γ_social_ are likely to downweight information relating to the past history of reward/social outcomes, and upweight information relating to current reward magnitudes.

### Mathematical model description

The model takes estimates of the probability of receiving good advice (*p*_*social,i*_) and the probability of green being rewarded (*p*_*green,i*_) at trial *i*, estimated via a Bayesian reinforcement learning optimized for adapting behavior depending upon the underlying volatility of the environment [see Figures 1B,C for graphs of tracked probabilities; for details of probability-tracking problem see (Behrens et al., 2007)]. These probability estimates are converted into *subjective* probabilities using the following transforms:

(1) $${\hat{p}}_{\text{social}},\;i=\frac{1}{1+e^{-\gamma_{\text{social}}\left(p_{\text{social}},i-0.5\right)}}$$

(2) $${\hat{p}}_{\text{green}},\;i=\frac{1}{1+e^{-\gamma_{\text{reward history}}\left(p_{\text{green}},i-0.5\right)}}$$

These subjective probabilities are then converted into an overall subjective probability of green yielding reward, q_i_:

(3) $${\hat{q}}_{i}=\frac{{\hat{p}}_{\text{social}},\;i{\hat{p}}_{\text{green}},i}{{\hat{p}}_{\text{social}},\;i{\hat{p}}_{\text{green},i+\left(1-{\hat{p}}_{\text{social}},i\right)\left(1-{\hat{p}}_{\text{green}},i\right)}}$$

if the partner suggests green on trial *i*, and

(4) $${\hat{q}}_{i}=\frac{{\hat{p}}_{\text{social}},\;i\left({\hat{p}}_{\text{green},\text{i}}\right)}{{\hat{p}}_{\text{social}},i\left(1-{\hat{p}}_{\text{green}},\;i\right)+\left(1-{\hat{p}}_{\text{social}},i\right){\hat{p}}_{\text{green},i}}$$

if the partner suggests blue.

The overall expected value of each option is then calculated as:

(5) $$V_{\text{green},i}=\hat{q}r_{\text{green},i}$$

and

(6) $$V_{\text{blue},i}=(1-\hat{q})r_{\text{blue},i}$$

where *r*_green_,*i* and *r*_blue_,*i* are the number of points available on green and blue options, respectively, on trial *i*. Finally, the probability of choosing the green option at trial *i* is calculated via a softmax function (O'Doherty et al., 2004):

(7) $$P\left(C_{i}=\text{green}\right)=\frac{1}{1+e^{-\beta (V_{\text{green}}-V_{\text{blue}})}}$$

and

(8) $$P\left(C_{i}=\text{blue}\right)=1-P(C_{i}=\text{green})$$

where β is an additional, third free parameter that determines the stochasticity of choice behavior.

We then used this model to estimate the log-likelihood of the observed data, at given values of the parameters

γ_reward history_, γ_social_, and β:

(9) $$\begin{array}{l} LL\text{​}\left(\gamma_{\text{social}},\gamma_{\text{reward history}},\beta \right)=\sum_{i}\text{log}[P(C_{i}=c_{i}|\gamma_{\text{social}},\\ \;\gamma_{\text{reward history}},\beta )] \end{array}$$

where *c_i_* denotes the option chosen by the subject on trial *i*. We custom-implemented a Bayesian estimation procedure in MATLAB (MathWorks, MA) to obtain the best-fitting parameters γ_social_, γ_reward history_ and β. Specifically, we performed direct numerical integration over the likelihood function of the observed data given the three free parameters. A grid of all possible parameter values of interest was formed, and we evaluated the likelihood of the data at each point in the grid, and then used marginalization to calculate the marginal likelihood of each parameter. All parameters were allowed to take values between 0.01 and 10, and the grid for numerical integration was evaluated in log space. This approach was selected because it gave a direct measure of the uncertainty associated with each parameter (i.e., the variance of each parameter's posterior distribution), in order to assess the reliability of model fitting.

### Relating fitted model parameters to variations in traits

The key question addressed here is which psychopathy-related traits are linked to the *between-subject variation* in the degree to which each optimally-tracked source of information is used to guide behavior, which is indexed in the model by the free parameters γ_reward history_ and γ_social_. To test this, we conducted two separate optimal scaled variable selections using the CATREG module in SPSS. This was done in order to establish the subscales of the PPI with the highest contributions in explaining the variance of each free parameter. For optimal scaling, all variables were defined on a numeric scale and discretized using a multiplication method, which transforms the variables into z-scores and multiplies them by 10. Two models were created which included all subscales of the PPI and the estimates for γ_reward history_ and γ_social_, respectively. Subsequently, variable selection with *lasso* [least absolute shrinkage and selection operator; (Tibshirani, 1996)] regularization was implemented to identify the “optimal” model for each free parameter. The optimal model was taken to be the model with the lowest expected prediction error and thus the highest accuracy given the data. This approach relies on shrinking the sum of the model coefficients by adding penalty terms to the model, resulting in coefficients that represent independent contributions of each variable as well as better model accuracy (Hartmann et al., 2009). For the regularization, the minimum of the standardized sum of squares was set at 0.0 and the maximum at 1.0 with a 0.02 increment in shrinkage at each step. This procedure yields an optimal model, which is the model with the smallest predicted margin of error. The latter was estimated with 0.632 bootstrapping (100 samples) (Efron and Tibshirani, 1993).

One advantage of this selection approach is that it overcomes a lot of the limitations of variable selection when using traditional stepwise regression analyses, such as the need for normality of variables (Hartmann et al., 2009), the related loss of power due to lack of compliance with assumptions, and the need for multiple comparison corrections associated with frequentist testing. After selection of the optimal model for each computational parameter, Pearson correlations were calculated between the scales in each model and the corresponding computational parameter in order to establish whether these covary. The significance of the correlations was tested with a non-parametric bootstrapping procedure (10.000 samples) to determine the confidence interval (CI) of each of the scales resulting from the variable selection procedure. If a correlation is significant its CI should not include the value of exactly 0. Thus, *both* the upper and lower bound of a CI should be either larger or smaller than 0.00. Finally, the oversampling procedure might have led to an atypical/non-normal distribution of the total and the scale scores of the PPI. Although our methodological approach did not rely on classical testing procedures requiring compliance with the assumption of normality, we still conducted Kolmogorov-Smirnov (KS) tests of normality to check whether the distribution of the total and scale scores of the PPI in the experimental sample was normal.

## Results

### General test of performance

First, we carried out an initial check to ascertain that participants were learning and were engaged in the task by comparing the amount of points earned at the end of the task with chance level performance. The results showed that the average amount of points earned (Mean = 10.372, *SD* = 780) was significantly higher than the amount that could be earned by guessing the correct choice on each trial (Mean = 7.292, *SD* = 577; *t*_(35)_ = 20.3, *p* < 0.001), indicating above chance performance and that participants were actively engaged in the task. Next, we also checked that the model provided a robust and reliable description of subject behavior. We found that the model, after parameter fitting, accurately predicted which of the two options subjects would choose on 80.6 ± 7.2% [mean ± standard deviation (SD)] of trials, indicating that it provided a robust description of subject behavior. Moreover, the uncertainty of estimated parameters (the SD of the posterior distribution) was relatively small compared to the magnitude/range of the estimated parameters (Mean γ_reward history_ = 1.14, SD range = 0.21–1.1; Mean γ_social_ = 2.18, SD range = 0.18–1.27), indicating that parameter fitting was reliable.

### Variable selection

**Figure 3:**
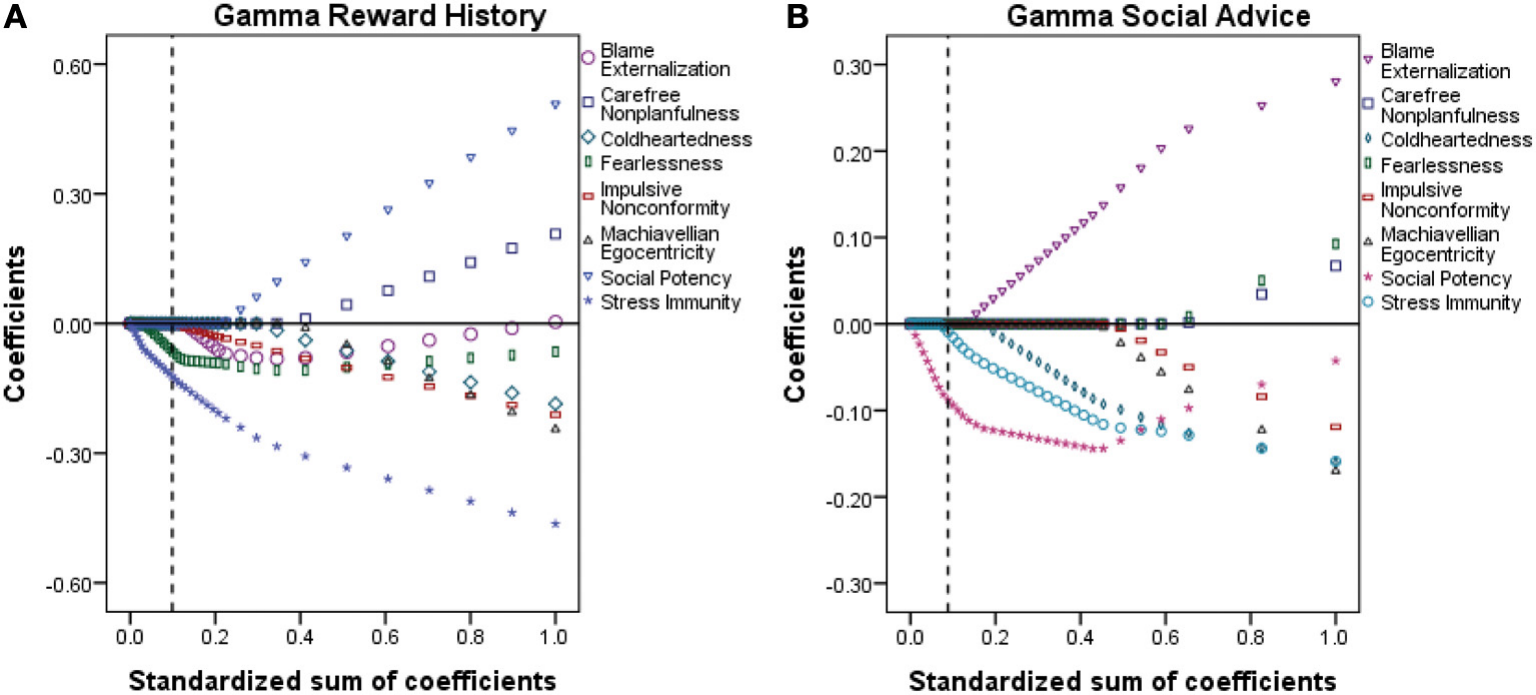
**Results of the variable selection procedure for γ_reward history_ (A) and γ_social_ (B)**. The maximum standardized sum of coefficients (SSC; x-axis) was set at 1.0, representing 100% of the contribution of the PPI scales to the corresponding γ parameter. Each sub-figure should be read from right (SSC = 1.0) to left (SSC = 0.0). The variable coefficients (y-axis) are displayed for different stages of shrinkage of the SSC. For each analysis, the variables included in the optimal model (i.e., the model with the lowest expected prediction error) are indicated with the vertical dashed line.

Here, we present the results of the two variable selection procedures run after the estimation of γ_reward history_ and γ_social_, which are displayed in Figure 3. The initial model is depicted at the far right of each panel. The systematic shrinkage of the standardized sum of coefficients forces the coefficients toward zero and for each step the resulting model is depicted to the left of the previous model. In both panels, the dashed vertical line indicates the optimal model. Note that for our purpose of solely identifying variables with the greatest contribution to the computational parameters, the magnitude and significance of the variable coefficients (indexed on the Y-axis) are of less interest and that the results do not warrant statistical significance in subsequent tests. Stress Immunity and Fearlessness were the traits that had the largest contributions to the variability across subjects of γ_reward history_ (Figure 3A). In contrast, the optimal model for γ_social_ included the variable Stress Immunity and Social Potency (Figure 3B).

### Correlations

**Figure 4:**
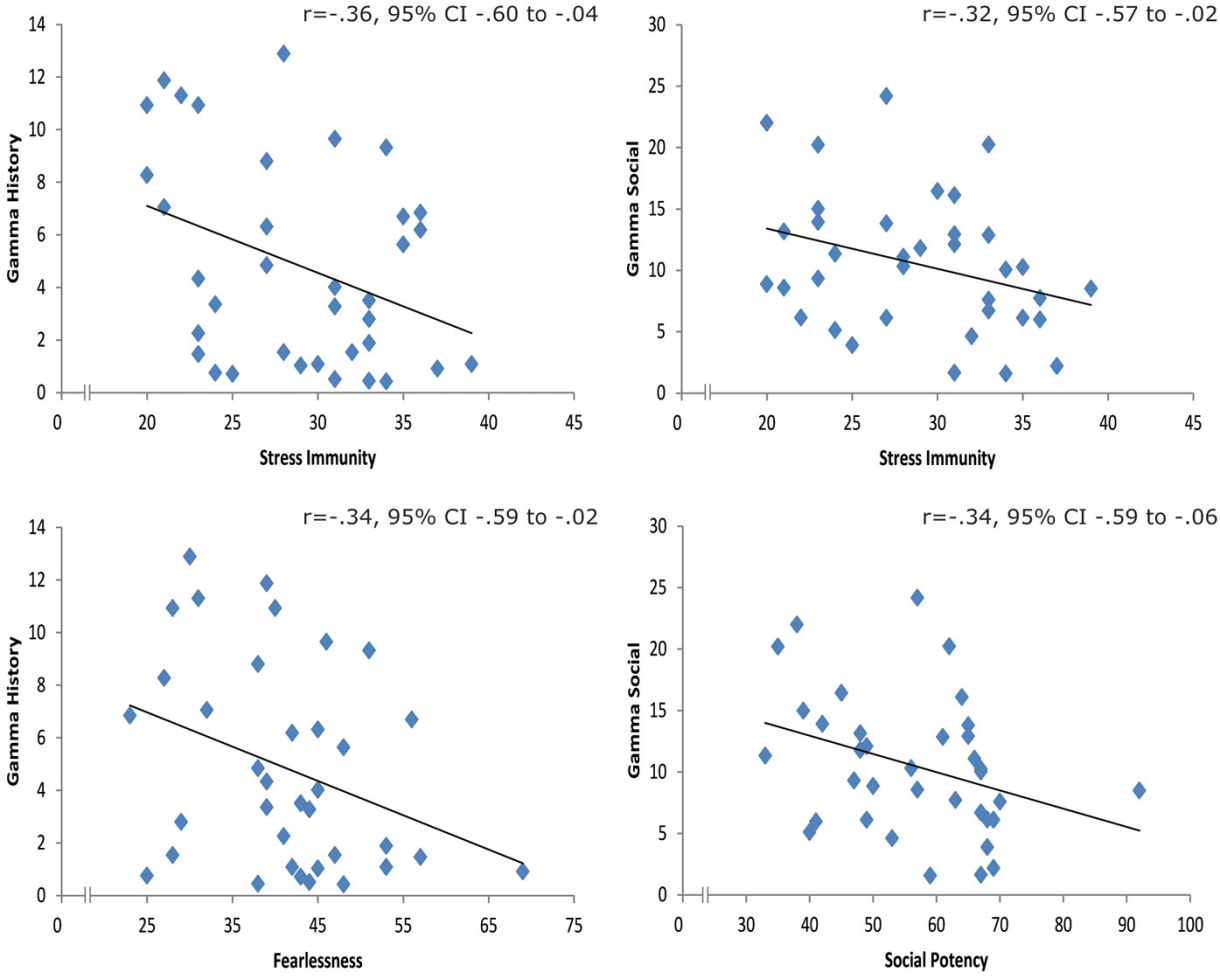
**Left:** scatterplots for the correlations between γ_reward history_ and Stress Immunity **(top left)**/Fearlessness **(bottom left)**. **Right**: scatterplots for the correlations between γ_social_ and Stress Immunity **(top right)**/Social Potency **(bottom right)**.

Subsequent correlation analyses yielded significant negative correlations between γ_reward history_ and Stress Immunity (*r* = −0.36, 95% CI −0.60 to −0.04) and γ_reward history_ and Fearlessness (*r* = −0.34, 95% CI −0.59 to −0.02). The correlation analyses revealed a negative relationship between γ_social_ and Social Potency (*r* = −0.34, 95% CI −0.59 to −0.06) and for γ_social_ and Stress Immunity (=−0.32, 95% CI −0.57 to −0.02). Thus, specific traits were related to different computational parameters quantifying individual difference in the use of reward and social information (see Figure 4).

### Additional tests

#### Additional correlation analyses

In order to demonstrate that the two computational parameters were uncorrelated and that the traits identified were uniquely related to either γ_social_ (Range = 1.57–24.2) or γ_reward history_ (Range = 0.42–12.9), we additionally examined the correlations between (1) γ_social_ and γ_reward history_, (2) Stress Immunity and Fearlessness with γ_social_ and (3) Social Potency with γ_reward history_. As expected, the computational parameters were not significantly correlated (*r* = 0. 11, 95% CI −0.18 to 0.58). Fearlessness was uncorrelated with γ_social_ (*r* = −0.10, 95% CI −0.44 to 0.33), as was Social Potency with γ_reward history_ (*r* = −0.07, 95% CI −0.35 to 0.25). These results indicate contributions of the different traits to the explained variance of the estimated model parameters. The tests of normality showed that the oversampling of the distribution tails in the selection pool (*N* = 485) did not cause the PPI measures in the experimental sample (*n* = 36) to deviate from normality (all KS-Z ≤ 0.95, *p*'s ≥ 0.33).

#### Comparison with an alternative computational model

Finally, we addressed concerns that our results may be a consequence of a use of a particular model, as opposed to a sensitive measure of the use of social information. We ran a direct comparison of a model that uses the Bayesian probability-tracking scheme and a Rescorla-Wagner learning model that has free parameters for learning rates (social and non-social). The correlation coefficient between γ_social_ for the Bayesian model, and γ_social_ for the fixed learning rate model, was 0.84; the correlation coefficient between γ_reward history_ for the Bayesian model, and γ_reward history_ for the fixed learning rate model, was 0.79. Thus, the fit parameters were not heavily influenced by the specific reinforcement learning model used, indicating that the results reported above paper are robust to the precise formulation of the RL model.

However, we elected to use the Bayesian RL model in the analysis above, because comparisons of model evidence vastly favored the Bayesian model. In 32 out of 36 subjects, the Bayesian Information Criterion favored the model with the Bayesian learning rate [paired *T*-test between BICs: *T*_(35)_ = 5.45, *p* < 0.000005 in favor of Bayesian model]. Similarly, in 25 out of 36 subjects, the Akaike Information Criterion, which has a smaller penalty than BIC for models with more free parameters (such as the fixed learning rate model), still favored the model with the Bayesian learning rate.

## Discussion

### Main findings

The present study is the first to use formal computational modeling to quantify how information from different sources is used during associative learning in order to provide evidence that variations in personality traits linked to psychopathy are differentially related to diminished use of social and reward information. This was achieved by establishing which specific traits related to psychopathy covary with the ability to actively use social and reward information to guide behavior as indicated by a computational model's parameter fits based on each individual participant's data. In this way, we succeeded in quantifying latent variables that cannot be observed overtly using traditional experimental approaches (Mars et al., 2012), and were able to relate these to personality traits proposed to be associated with core aspects of the construct of psychopathy.

We found that the extent to which participants tended to use reward and social information was related to different personality traits. Traits capturing lack of anxiety (Stress Immunity) and lack of fear (Fearlessness) were negatively correlated with the extent to which previous reward history was used to make decisions. The use of social information was found to have a negative relationship with participants' perceived ability to charm and manipulate others (Social Potency) and lack of anxiety. Importantly, our effects are selectively associated with personality traits argued to be central to psychopathy, while none of the traits more related to externalizing personality styles were substantially linked to the computational parameters in the present study. In other words, the results suggest that the deficient use of reward and social information during learning could be specific to psychopathic personality styles rather than general antisociality, and also that the deficient implementation of information that seems to be present in male offenders diagnosed with a psychopathic disorder translates to common personality traits linked to psychopathic tendencies in the non-clinical female population.

### Comparison with previous work

The use of previous reward history was negatively correlated with scores on Stress Immunity and Fearlessness. These findings converge with evidence relating both low anxiety and low fear to disturbed associative learning in clinical psychopathy (Arnett et al., 1993; Birbaumer et al., 2005). Particularly, work by Newman and colleagues has shown that disturbed passive avoidance learning is predominantly found in individuals with psychopathy with low trait anxiety relative to those with high anxiety (Newman et al., 1990; Arnett et al., 1993). Similarly, psychopathic behavior has also repeatedly been linked to reduced fear reactivity in both clinical and non-clinical samples (Patrick et al., 1993; Blair et al., 2002; Benning et al., 2005; Jones et al., 2009) and, importantly, impaired fear-conditioning (Flor et al., 2002; Birbaumer et al., 2005). The central premise here is that aversion to negative outcomes induces a negative affective state such as fear/anxiety, which is in turn associated with the actions/contexts that lead to these negative affective states. With respect to psychopathy, it has been proposed that a low propensity to experience these negative affective states plays a role in the formation of weak associations with events leading to negative outcomes and thus contribute to an impairment in the process of associative learning (Blair, 2005). Our results add support to this notion by pointing out that increased trait fearlessness and lack of anxiety contribute to reduced use of information to guide behavior during associative learning.

One important consideration is that in tasks using behavioral performance as an index for associative learning, these outcome measures not only represent the integrity of the associative process (i.e., the linking sensory events to outcomes) but also the individual's ability to integrate and use relevant sensory information to initiate and execute motor responses/observable behavior (Daunizeau et al., 2010). Thus, covert behavior is the integrated end-result of various processing steps in different domains. Therefore, impaired performance could reflect deficient processing in the sensory domain (e.g., the establishment of associations/learning), or in the motor domain (e.g., execution errors), or maybe a problem in the interaction between the sensory domain and the motor domain (e.g., using learned associations as input to guide motor responses). The present findings indicate that trait fear and anxiety play an important role in the active implementation of available information to guide changes in behavior. This suggests that impairments in associative learning previously found in clinical psychopathy might also be (partly) due to a deficiency in *using* reinforcement information appropriately to drive behavior, which, depending upon the experimental paradigm used, may ultimately manifest itself as disturbed learning.

The use of information provided by the confederate, i.e., the use of social information history, was found to have a negative relationship with participants' perceived ability to charm and manipulate others (Social Potency) and their level of trait anxiety (Stress Immunity). Social Potency and anxiety encompass behavior relevant for social functioning. High Social Potency is commonly associated with social dominance and one's belief that one is able to successfully manipulate others. We could hypothesise that people who believe that they can manipulate others are more likely to believe that others will try to manipulate them, when *mentalizing* about the likely intentions of the social partner (Behrens et al., 2008; Hampton et al., 2008; Chang et al., 2011). That is, these individuals may be more likely to engage in making inferences about what others may think we believe, i.e., second-order beliefs. A possible explanation for the relationship between lack of anxiety and use of social advice could be that as trait anxiety decreases, individuals experience less anxiety evoked by the potential negative consequences of discarding the confederate's advice. Thus, as individual levels of trait anxiety decrease, not using social advice might be experienced as less aversive, in a way similar to reward-based learning. This prediction would be in line with findings showing that associative learning of social and non-social information follow the same mechanistic principles (Behrens et al., 2008). In sum, our results suggest that reduced anxiety and second-order belief systems might play an important role in explaining social cognition in psychopathy. Future studies should focus on mapping how second-order beliefs are related to general traits relevant to psychopathy in the general community as well as in offenders with a clinical diagnosis of psychopathy.

### Interpretational limitations

This is one of the first studies that has attempted to link scores on psychopathy-related personality traits with latent variables from a computational model that was fit to each participant's behavior (see also White et al., 2013). This approach has been suggested to have tremendous potential in the study of psychopathology and in psychiatry in general, as it has the potential to be able to disentangle separate aspects of complex multidimensional syndromes (Montague et al., 2012). However, this does not mean that the approach is not without its limitations. Below we suggest some potential improvements and avenues for future studies.

One potential caveat is that in our current model the learning rates for reward and social information were not allowed to vary across subjects. This is due to limitations in the number of trials we would need to reliably estimate more free parameters. Instead, the model used (Behrens et al., 2007) was one that adapts its learning rate dependent upon the current level of volatility in the environment. In the current study, we instead set out to test the hypothesis that the use of different types of information is related to different personality traits that are relevant for psychopathy. The present study included a sample of healthy individuals and previous studies have shown that healthy individuals are able to estimate the volatility of the environment and adapt their learning rate accordingly, and that this behavior is reproduced reliably by our computational model (Behrens et al., 2008). Future computational studies could be designed to explicitly test the hypothesis that it is use of information rather than (only) learning rate in general that is impaired in offenders diagnosed with psychopathic disorder according to the Psychopathy Checklist-Revised (PCL-R) (Hare, 2003), as suggested by some of our previous findings (von Borries et al., 2010; Brazil et al., 2013).

Another potential limitation of our current study is the size of our group of participants. Although we have used a large sample of participants compared to most computational modeling studies (e.g., Nieuwenhuis et al., 2005; Behrens et al., 2008; Yoshida et al., 2008; Boorman et al., 2009; Mars et al., 2012; Brodersen et al., 2013), some may argue that it is on the lower side in studies in psychological research on personality. We have taken care to ensure the robustness of our effects through the methodology employed, but the size of our sample can still be raised as a criticism despite the fact that our methodology bypasses the need for compliance with the requirements of classical inferencing [for more details on the overlooked issues with various common beliefs about sampling and sample sizes we highly recommend (Friston, 2012, 2013)]. Furthermore, the fact that previous studies using our model found robust results even with much lower subject numbers is therefore quite reassuming (e.g., Behrens et al., 2008; Boorman et al., 2009).

Finally, our experimental sample consisted of female participants and it could be argued that the findings might not extent to the male population. However, previous studies in clinical psychopathy suggesting deficient use of information to adapt behavior included only male participants (Brazil et al., 2009, 2013; von Borries et al., 2010) and as the current results converge with those obtained in male-only samples they support the notion that this particular deficiency in using information to guide behavior does not seem gender-specific. In support of this claim, recent studies on the relationship between psychopathic traits in community samples, empathic responding and moral processing suggest a similar relationship in both males and females (Seara-Cardoso et al., 2012, 2013). Interestingly, Seara-Cardoso et al. (2013) found a negative relationship between these cognitive functions and the interpersonal-affective traits in females. In this study they used a different operationalization of psychopathy (Paulhus et al., 2013) and assessed other aspects of cognitive functioning relative to the present study, but the findings are in line with ours in that they point out that gender might not have an overall impact on the link between psychopathy-related traits and certain aspects of cognition.

## Conclusions

The present study is the first to directly assess the relationship between variations in psychopathy-related personality traits and the amount of information that is used during associative learning of social and reward information. The findings show that the use of both types of information to guide behavior decreases as the presence of personality traits proposed to be related to the interpersonal-affective aspect of psychopathy increases. More specifically, lower trait anxiety and fearlessness were associated with reduced use of one's reinforcement history and an increased perceived ability to manipulate others and reduced anxiety were related to diminished use of social advice. Additionally, the findings suggest an extension of results obtained in male offenders with clinical psychopathy to the general (female) population by showing that the newly-discovered latent variables are linked to variations in personality traits that are important for the construct of psychopathy. Importantly, however, it still remains to be investigated whether these computational parameters can account for some of the impairments in adaptive behavior found in forensic psychiatric populations with a psychopathic disorder. The results illustrate the potential advantages of employing formal models to discover computational phenotypes in clinical populations (Montague et al., 2012), as well as their usefulness in gaining more insight into the exact personality traits related to the cognitive deficiencies observed in many personality disorders. The present findings might also have implications for treatment aimed at altering behavior, as the success of treatment partly relies on the patient's ability to incorporate and use information from past experience as well as information provided by therapists.

### Conflict of interest statement

The authors declare that the research was conducted in the absence of any commercial or financial relationships that could be construed as a potential conflict of interest.
